# Direct Growth of Highly Conductive Large‐Area Stretchable Graphene

**DOI:** 10.1002/advs.202003697

**Published:** 2021-02-01

**Authors:** Yire Han, Byeong‐Ju Park, Ji‐Ho Eom, Venkatraju Jella, Swathi Ippili, S. V. N. Pammi, Jin‐Seok Choi, Hyunwoo Ha, Hyuk Choi, Cheolho Jeon, Kangho Park, Hee‐Tae Jung, Sungmi Yoo, Hyun You Kim, Yun Ho Kim, Soon‐Gil Yoon

**Affiliations:** ^1^ Department of Materials Science and Engineering Chungnam National University Daeduk Science Town Daejeon 34134 Republic of Korea; ^2^ P&T Division SK Hynix Cheongju 28433 Republic of Korea; ^3^ Analysis Center for Research Advancement (KARA) Korea Advanced Institute of Science and Technology 291 Daehak‐ro, Yuseong‐gu Daejeon 34141 Republic of Korea; ^4^ Advanced Nano‐Surface Group Korea Basic Science Institute (KBSI) 169‐148 Gwahangno, Yuseong‐gu Daejeon 34133 Republic of Korea; ^5^ Department of Chemical and Biomolecular Engineering Korea Advanced Institute of Science and Technology Daejeon 34141 Republic of Korea; ^6^ Advanced Materials Division Korea Research Institute of Chemical Technology Daejeon 34114 Republic of Korea; ^7^ Department of Chemical Convergence Materials and Processes KRICT School University of Science and Technology Daejeon 34114 Republic of Korea

**Keywords:** giant domain size, high conductivity, low‐temperature growth, superb stretchability, transfer‐free monolayer graphene

## Abstract

The direct synthesis of inherently defect‐free, large‐area graphene on flexible substrates is a key technology for soft electronic devices. In the present work, in situ plasma‐assisted thermal chemical vapor deposition is implemented in order to synthesize 4 in. diameter high‐quality graphene directly on 10 nm thick Ti‐buffered substrates at 100 °C. The in situ synthesized monolayer graphene displays outstanding stretching properties coupled with low sheet resistance. Further improved mechanical and electronic performances are achieved by the in situ multi‐stacking of graphene. The four‐layered graphene multi‐stack is shown to display an ultralow resistance of ≈6 Ω sq^−1^, which is consistently maintained during the harsh repeat stretching tests and is assisted by self‐*p*‐doping under ambient conditions. Graphene‐field effect transistors fabricated on polydimethylsiloxane substrates reveal an unprecedented hole mobility of ≈21 000 cm^2^ V^−1^ s^−1^ at a gate voltage of −4 V, irrespective of the channel length, which is consistently maintained during the repeat stretching test of 5000 cycles at 140% parallel strain.

## Graphical abstract

Averaged mobility of 42 GTO‐FET (field‐effect transistor) devices indicates high hole and electron mobilities of 21 000 and 12 000 cm^2^ V^−1^ s^−1^, respectively, at *V*_GS_ = ± 4 V. The Ti–O–C interfacial layer indicated by the density functional theory‐predicted GTO model produces the unprecedented stretchability of the GTO‐FETs during 5000 cycles of parallel stretching of up to 140% strain.

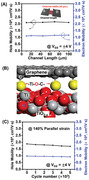

The leading edge of soft electronics research is driving toward the realization of large‐area and stretchable transparent electrodes and devices that integrate with each other into delicate soft displays, sensors, or energy harvesters.^[^ ^1^, ^2^, ^3^, ^4^, ^5^, ^6^, ^7^, ^8^, ^9^, ^10^, ^11^, ^12^ ^]^ In particular, graphene satisfies most of the critical requirements for flexible electrode and device applications and has attracted significant attention.^[^ ^13^, ^14^, ^15^ ^]^ However, the high operation temperature of at least 1000 °C required for the large‐area growth of graphene byconventional chemical vapor deposition (CVD) process hinders the widespread application of graphene‐based electrodes and devices. Moreover, the conventional two‐step process consisting of high‐temperature CVD‐based growth of graphene on metallic substrates and its subsequent transfer onto flexible substrates inevitably generates wrinkles, ripples, and metallic residues that significantly lower the graphene quality.^[^ ^16^, ^17^, ^18^, ^19^, ^20^, ^21^ ^]^ Even the most recent and advanced CVD‐based graphene growth technique demonstrates that the synthesis of large‐area graphene via industrially friendly and versatile CVD is challenging.

The present authors have previously proposed a possible transfer‐free CVD‐based graphene growth technology by combining the direct current sputtering of a Ti‐buffer layer onto the substrate with subsequent CVD growth of graphene on the Ti.^[^ ^22^ ^]^ Based on the results of post‐synthesis analysis and density functional theory (DFT) calculation, the strong Ti–C interaction was proven to generate the required carbon seeds on the Ti and to facilitate the thermodynamically driven spontaneous growth of a graphene monolayer at 150 °C. In previous report, DC‐sputtered Ti‐buffered substrates were moved to the separate CVD system under ambient air for direct graphene synthesis. Because Ti‐buffer layer was naturally oxidized under ambient conditions, the oxidized Ti‐buffer layer was reduced to micrometer size of a clean Ti for various holding times under a reduction atmosphere of H_2_ at 150 °C. Therefore, the graphene area was limited within the micrometer scale because the graphene grows only on the clean Ti. These results demonstrated the feasibility of the CVD‐based method for the low‐temperature growth of graphene, thus potentially motivating technological advances in relevant fields. However, the high sheet resistance (≈600 Ω sq^−1^) and narrow size (20 µm × 20 µm) of the graphene synthesized on Ti require further advances to enable the low‐temperature growth of high‐quality, large‐area graphene, which would be applicable to practical soft electrodes and electronic devices.

In the present work, a two‐step plasma‐assisted thermal chemical vapor deposition (PATCVD) method is developed, which enables the in situ growth of a Ti‐buffer layer onto a target substrate and the subsequent low‐temperature growth of monolayer graphene (*m*Gr) on the Ti. The transparent 4 in. diameter *m*Gr directly grown onto the 10 nm thick Ti‐buffered polyethylene terephthalate (PET) and polydimethylsiloxane (PDMS) substrates at 100 °C is shown to exhibit unprecedented stretchable properties combined with an extremely low sheet resistance of 80 Ω sq^−1^. Moreover, the in situ deposition of multiple *m*Gr layers onto PDMS is shown to further improve the stretchability and decrease the sheet resistance to 6 Ω sq^−1^, making this product suitable for direct integration into graphene‐based transparent electrodes or electronic devices. Finally, the field‐effect transistors (FETs) constructed from the PATCVD‐grown *m*Gr are shown to exhibit high stretchability along with unprecedented hole mobility of ≈21 000 cm^2^ V^−1^ s^−1^ at a gate voltage of −4 V, which is consistent over a wide range of channel lengths and under repeat stretching tests.

As shown schematically in **Figure** **1**A, the *m*Gr was reproducibly grown at temperatures below 150 °C on a Ti‐buffered substrate via PATCVD, which enables direct methane decomposition and spontaneous graphene growth. For example, the optical image in Figure 1B shows a 4 in. diameter *m*Gr that was successfully grown during 2 h at 100 °C. The sheet resistance of this *m*Gr is as low as 76–86 Ω sq^−1^, which is an improvement over the best literature‐reported value.^[^ ^23^ ^]^ Moreover, the temperature‐dependent Raman spectra of the *m*Gr layers presented in Figure 1C demonstrate that the *m*Gr was successfully synthesized above 80 °C. In addition, the consistency of the Raman spectra of *m*Gr samples grown on different Ti‐buffered substrates (Figure S1, Supporting Information) confirms that the Ti‐buffer layer controls the *m*Gr growth mechanism. The surface of the *m*Gr is visualized over 50 × 50 µm^2^ by the micro‐Raman mapping images in Figure 1D, with insets showing histograms of the intensity ratios *I*_2D_/*I*_G_ and *I*_D_/*I*_G._ The estimated intensity ratio values *I*_2D_/*I*_G_ = 2.1 ± 0.05 and *I*_D_/*I*_G_ = 0.01 ± 0.008 confirm the superior quality of the PATCVD‐grown *m*Gr. Moreover, the low *I*_D_/*I*_G_ is below the previously reported values for nanometer‐area graphene grown directly on SiO_2_ substrates via CVD at 800 °C (*I*_D_/*I*_G_ = 0.3) and for transferred single‐crystalline graphene grown on hydrogen‐terminated germanium at temperatures between 900 and 930 °C (*I*_D_/*I*_G_ <0.03).^[^ ^24^, ^25^ ^]^ In order to analyze the graphene quality without TiO_2−_ *_x_* layer, graphene grown on Ti‐buffered Cu foil was dry‐etched using the dry‐transfer process (Figure S2, Supporting Information). The complete removal of TiO_2−_ *_x_* layer was confirmed via X‐ray photoelectron spectroscopy (XPS) wide scan (Figure S3a, Supporting Information) and the etched graphene was transferred to the Cu grid for high‐resolution transmission electron microscope (TEM) analysis. Based on the high‐resolution TEM image (Figure S3b, Supporting Information) and a selected‐area electron diffraction pattern (inset of Figure S3b, Supporting Information), the monolayer graphene had an atomically thin, high‐quality graphene lattice. The transferred graphene revealed D peaks by defects formed during the transfer process (Figure S4a, Supporting Information) and showed an increase of sheet resistance as high as 100 ± 3 Ω sq^−1^ (Figure S4b, Supporting Information).

**Figure 1:**
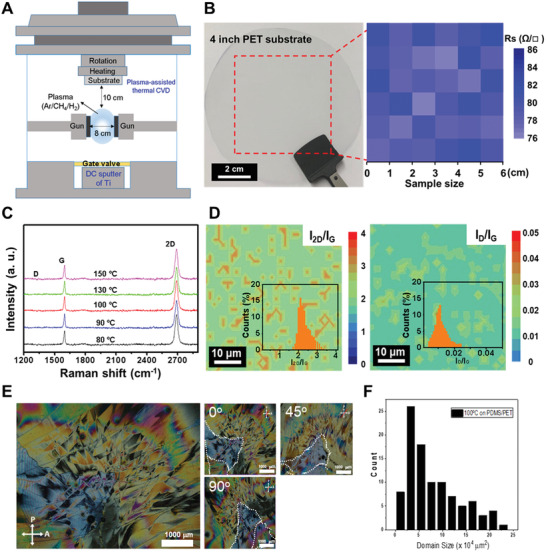
PATCVD‐grown *m*Gr on Ti‐buffered substrate. A) Schematic diagram of the assembly process using PATCVD and DC sputtering for Ti. B) Optical image of a 4 in. wide *m*Gr synthesized on a Ti‐buffered PET substrate at 100 °C and the corresponding resistance map of the area enclosed by the red dashed lines. C) Temperature‐dependent Raman spectra of the *m*Gr. D) Micro‐Raman mapping images observed in 50 × 50 µm^2^ area with insets presenting the histograms of the *I*_2D_/*I*_G_ and *I*_D_/*I*_G_ intensity ratios. E) POM images of the liquid crystal‐coated‐*m*Gr films synthesized on Ti (10 nm)‐buffered PDMS substrate (2 × 2 cm^2^ size) at 100 °C (left), and the effect of microscope‐stage rotation angle on the appearance of the domains (right). F) Domain size distribution of the *m*Gr grown at 100 °C. Domain size distribution was analyzed by measurements above 80 domains.

Because graphene is grown at high temperatures on various catalytic metals, graphene synthesis at low temperature on titanium has been considered an uncertain process because of the possible formation of titanium carbide on the titanium surface rather than graphene. To eliminate these apprehensions, graphene grown on titanium‐buffer layer at 100 °C was analyzed via XPS, and no titanium carbide was observed (red circle of Figure S5, Supporting Information), which demonstrated no formation of titanium carbide on a titanium surface at low temperature of 100 °C. The carbon was determined to be sp^2^‐bonded carbon, which revealed the graphene films (Figure S5, Supporting Information).

In the present work, although a metallic Ti‐buffer layer was used to grow the large‐area *m*Gr on a flexible substrate, the Ti layer was spontaneously and completely oxidized into semiconducting TiO_2−_ *_x_* as the PATCVD‐grown *m*Gr was exposed to ambient conditions. This is confirmed by the typical TiO_2−_ *_x_* feature in the X‐ray photoelectron spectra of the *m*Gr grown at 100 °C and subsequently exposed to air (Figure S6, Supporting Information; refer to the Supporting Information for more detailed results of the analysis). Therefore, the *m*Gr is supported on TiO_2−_ *_x_* and is referred to hereafter as GTO. Sufficient experimental evidence of metal oxidation in the case of CVD‐graphene on a Cu catalyst occurred when molecules penetrated the graphene grain boundary and diffused laterally along the graphene–metal interface.^[^ ^26^, ^27^, ^28^ ^]^ As indicated by the above‐mentioned results, this semiconducting TiO_2−_ *_x_* layer does not exert any negative effect on the conductivity of the PATCVD‐grown *m*Gr.

The distribution and size of the graphene domains synthesized directly on Ti (10 nm)‐buffered PDMS substrates at 100 °C were demonstrated by imaging the birefringence of a GTO surface covered with well‐known nematic liquid‐crystal molecules (5CB: 4‐pentyl‐4’‐cyanobiphenyl) in accordance with previous studies.^[^ ^29^, ^30^ ^]^ The polarized optical microscopy (POM) images in Figure 1E clearly distinguish the individual domains on the surfaces of the 5CB‐coated GTO by the retardation of colors that arises from the interference between two components of polarized light split by the anisotropic 5CB molecules. As the microscope stage was rotated during examination, the same colored domains revealed the same level of birefringence changes. When the 5CB‐coated GTO (0°, left image) was rotated counterclockwise by 45° (center image) and 90° (right image), the domains indicated by the white dotted‐region in the three small images in Figure 1E were correspondingly rotated with no change in the domain shape.

Previous studies on the growth of *m*Gr on a Ni catalytic metal have shown the grain size to be a positive function of the growth temperature and to be highly dependent on the Ni grain size due to the surface‐reaction‐controlled mechanism (activation energy of 0.52–0.77 eV).^[^ ^31^, ^32^ ^]^ Based on the distribution of graphene domains observed in the POM image (Figure 1F), an average domain size of ≈266 µm was observed in the graphene films synthesized on Ti (10 nm)‐buffered PDMS substrates at 100 °C. The Arrhenius plot of the *m*Gr growth rate (Figure S7, Supporting Information) reveals a significantly decreased growth activation energy of 0.28 eV, which is a distinctive feature of a mass‐transport‐controlled thin‐film growth mechanism,^[^ ^33^ ^]^ which demonstrated a large domain size of monolayer graphene grown at low temperature. Improvement of growth rate can be achieved by a sufficient supply of carbon source because our graphene growth was controlled by a mass‐transport mechanism.

Although graphene is expected to find wide application in flexible electronic devices,^[^ ^20^, ^23^ ^]^ its inherent mechanical and electronic superiority are diminished by conventional CVD and transfer methodology because structural defects are generated upon transfer and the transferred graphene cannot strongly adhere to the flexible substrate. Because graphene has a strong carbon–carbon in‐plane network, the upper stretching strain limit at which the CVD‐grown graphene transferred onto a PDMS can recover its initial conductivity is only 6%.^[^ ^20^ ^]^ Moreover, the initial conductivity of conventional PDMS‐supported graphene under an applied strain perpendicular or parallel to the current flow is maintained only at less than 11% strain.^[^ ^20^ ^]^ By contrast, since the PATCVD technique used in the present work allows the in situ deposition of GTO on a stretchable substrate, enhanced stretchability of the GTO is anticipated.

The results presented in **Figure** **2**A demonstrate remarkable control of both the perpendicular and parallel normalized resistance change ((*R* − *R*_o_)/*R*_o_) at values of less than 10 for stretching strains of up to 60% for a 70 µm × 15,000 µm GTO strip grown onto a 3.0 cm × 4.0 cm PDMS substrate. Moreover, even at 70% strain, the perpendicular (*R* − *R*_o_)/*R*_o_ was controlled at around 10, which is the general upper limit for graphene stretching‐resistance tests.^[^ ^34^, ^35^ ^]^ In addition, an LED switching test was performed to visually demonstrate the outstanding performance of the GTO/PDMS as a stretchable electrode, as shown in Figure 2B and Video S1 in the Supporting Information. Although the illuminance (lx) versus stretching strain curve in Figure 2B is seen to gradually decrease at greater than 30% strain, no flickering is observed in Video S1 in the Supporting Information for up to 60% strain. Moreover, even when stretched once up to 90% strain, the released GTO recovered 93% of its initial illuminance, thus demonstrating the excellent mechanical consistency of the as‐prepared GTO.

**Figure 2:**
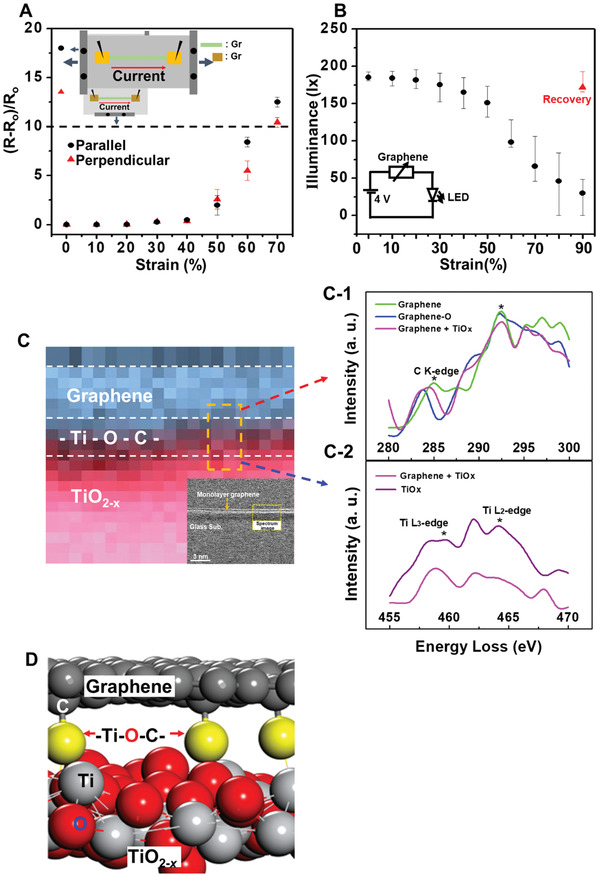
Mechanical and electronic superiority of the GTO. A) The change in normalized resistance, (*R* − *R*_o_)/*R*_o_, of the *m*Gr due to stretching parallel (black circles) and perpendicular (red triangles) to the current directions. The stretchability test was performed using a 70 µm (width) × 15 000 µm (length) GTO strip grown onto a 3.0 cm × 4.0 cm PDMS substrate. B) Plot of the commercial green light‐emitting diode (LED, Luckylight Electronics Co. China) intensity (illuminance) against *m*Gr stretching strain. The red triangle indicates the LED light intensity measured after release of the *m*Gr from 90% strain. C) Cross‐sectional EELS mapping image of the *m*Gr (pixel size = 0.05 nm) revealing the oxidized TiO_2−_ *_x_* and the bridging oxygen layer. The inset at the bottom right is the low‐resolution annular dark‐field transmission electron microscope image with the corresponding area indicated by yellow dashed lines. C‐1,C‐2) EELS spectra of C K‐edge and Ti‐L edge observed from the graphene and TiO_2−_ *_x_* layer. D) DFT model of the *m*Gr linked to TiO_2−_ *_x_* by bridging oxygen atoms (highlighted in yellow).

The breaking strength of polycrystalline graphene has been shown to be a positive function of the domain size.^[^ ^36^ ^]^ For example, Lee et al. reported breaking strengths of ≈91 and 118 GPa for graphene with domain sizes of ≈2.5 and 200 µm, respectively.^[^ ^37^ ^]^ Hence, because the domain size of the GTO samples prepared in the present work is generally larger than 260 µm (Figure 1E,F), their high stretchability is partially attributed to the domain size effect.

The cross‐sectional electron energy loss spectroscopy (EELS) mapping image and spectra (C K‐edge and Ti L‐edge) of the GTO in Figure 2C clearly distinguish the thin layer of oxygen ions bridging the *m*Gr and TiO_2−_ *_x_*. This chemical structure is also indicated by the DFT‐predicted GTO model system in Figure 2D.

As indicated by Figure S8 in the Supporting Information, the area‐normalized DFT‐calculated interaction energy (*E*_int_) was reduced from −11.5 eV nm^−2^ between *m*Gr and Ti to −0.4 eV nm^−2^ between the *m*Gr and TiO_2−_ *_x_* of the GTO model. Taken together, the EELS and DFT results suggest that the GTO is a layered composite material consisting of *m*Gr floating on TiO_2−_ *_x_*.

The interface between *m*Gr and TiO_2−_ *_x_* can positively contribute to the stretchability of GTO. The stretching mechanism of graphene has been studied by Bagchi et al. via DFT calculations to find that the critical shear strength for sliding of the graphene layers was significantly decreased from 5.27 ± 0.46 GPa on Ti to 0.07 ± 0.015 GPa TiO_2−_ *_x_* due to the weaker interaction between graphene and TiO_2−_ *_x._* ^[^ ^38^ ^]^ In the present work, the contribution of the interfacial sliding effect to the high stretchability of GTO was experimentally elucidated by transferring the *m*Gr layer of the GTO onto a PDMS substrate and examining how the (*R* − *R*_o_)/*R*_o_ responds to the parallel strain. As indicated in Figure S9 in the Supporting Information, the transferred graphene revealed an extreme (*R* − *R*_o_)/*R*_o_ increase at 60% strain, thus confirming the advantage afforded by the graphene–TiO_2−_ *_x_* interfacial layer for improved stretchability of the GTO. Overall, these results demonstrate that the combined effects of domain size and facilitated graphene sliding at the *m*Gr‐TiO_2−_ *_x_* interface lead to the mechanical and electronic supremacy of the PATCVD‐grown GTO.

The resistance of the GTO was further reduced by the direct in situ stacking of up to four GTO layers at 100 °C on a flexible substrate, as shown schematically on the left‐hand side of **Figure** **3**A. Meanwhile, the transmittance measurements on single‐ and multi‐layered GTO (*n*GTO, where *n* is the total number of stacked GTO layers) and the corresponding photographic images (insets) presented on the right‐hand side of Figure 3A indicate that each additional GTO layer reduces the optical transmittance by ≈2.2%. This is close to the reduction rate observed in a transferred multilayer graphene system,^[^ ^23^ ^]^ thus confirming that the TiO_2−_ *_x_* layer has no significant influence on the transmittance. As shown in Figure 10 in the Supporting Information, the *m*Gr was preserved in each individual stacking layer, so that the *m*Gr layers of the *n*GTO were sandwiched between successive TiO_2−_ *_x_* layers.

**Figure 3:**
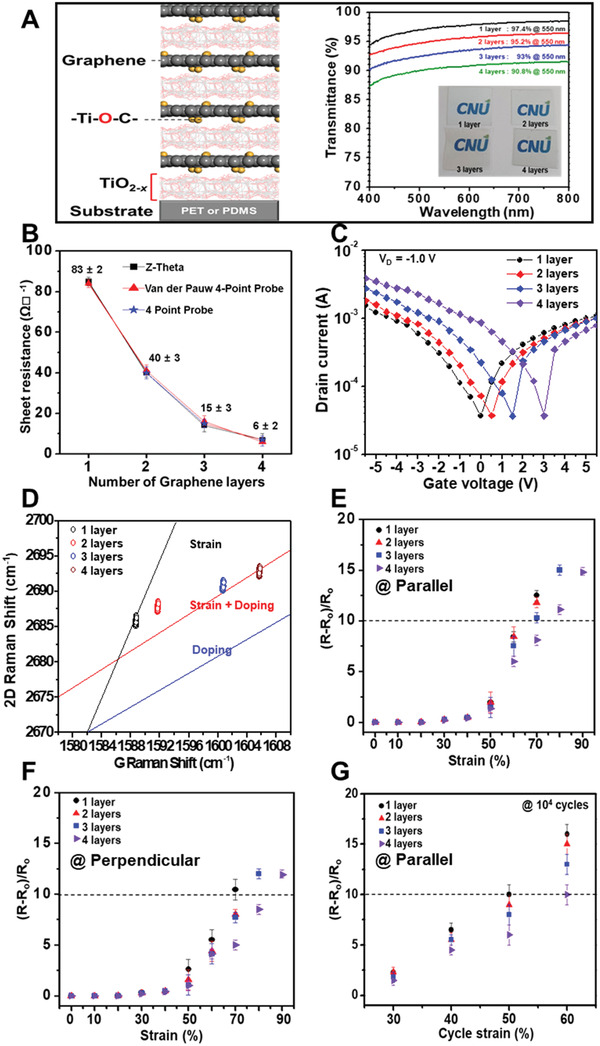
Improved mechanical and electronic performance of self‐*p*‐doped multi‐stacked GTO. A) Schematic cross‐sectional view of the 4GTO (left) and the transmittance‐wavelength profiles of the single‐layer and multi‐stacked *n*GTOs (*n* = 2, 3, or 4) shown in the inset photographs. The transmittance of the multilayer stacked graphene was measured via UV‐vis spectroscopy with a beam diameter of 0.8 cm. B) Plots of sheet resistance against number of layers in the *n*GTOs, as measured via the Z‐theta, van‐der‐Pauw, and four‐point probe methods using 2 × 2 cm^2^ sample size. C) Transfer characteristics of the multi‐stacked *n*GTO‐FETs with a channel width of 800 µm and a channel length of 200 µm. D) G‐2D Raman maps of the multi‐stacked *n*GTOs using 2 × 2 cm^2^ sample size. E,F) The (*R* − *R*_o_)/*R*_o_ ratios of the GTO and *n*GTOs measured by E) parallel and F) perpendicular strain to the current directions using the same dimension with Figure 2A. G) The (*R* − *R*_o_)/*R*_o_ ratios of the GTO and *n*GTOs measured after 10^4^ cycles of repeat stretching.

The Z‐theta, van‐der‐Pauw, and four‐point probe measurements presented in Figure 3B reveal a significantly decreased sheet resistance of 6 ± 2 Ω sq^−1^ for the 4GTO which, along with the high transparency of 90.8%, is superior to the previous best results. Even the lowest reported resistance of a transferred four‐layer graphene system (≈30 Ω sq^−1[^ ^23^ ^]^) is five times greater than that of the present 4GTO. This remarkable decrease in the resistance upon GTO multi‐stacking is attributed to the nondestructive charge transfer *p*‐doping by oxygen adsorbed on the *m*Gr of each stack, which is revealed by the gradually increasing positive shift in the Dirac voltage of the *n*GTO‐FETs in Figure 3C and by the G‐2D Raman map of the strained GTO in Figure 3D.^[^ ^39^ ^]^ By contrast, no notable Dirac shift was observed in the single‐layer GTO‐based FET, thus suggesting that *p*‐doping is induced by the TiO_2−_ *_x_* interlayer sandwiched between two *m*Gr layers in the GTO multi‐stack. In addition, the results in Figure S11 in the Supporting Information demonstrate that the self‐*p*‐doping effect of multilayer *n*GTO is not degraded by high‐temperature treatment.

Intuitively, a thick‐stacked multilayer *n*GTO is naturally more rigid than the single‐layer GTO. Nevertheless, Won et al. reported that transferred multilayer graphene displayed excellent stretchability due to the strain relaxation facilitated by sliding among the graphene layers, such that bi‐ or tri‐layered graphene stacked by a transfer process was stretchable by up to 30% strain.^[^ ^40^ ^]^ Indeed, the (*R* − *R*_o_)/*R*_o_ ratios obtained from the single‐cycle stretching test (Figure 3E,F) and the repeat‐cycle stretching test (10^4^ cycles, Figure 3G) performed in the present work demonstrate that multi‐stacking significantly improves the stretchability of the *n*GTO.

In detail, (*R* − *R*_o_)/*R*_o_ ratios of less than 10 were maintained for the 4GTO under single cycles of up to 70% and 80% strain parallel and perpendicular to the current direction, respectively (Figure 3E,F), and under 10^4^ repeat‐cycles of up to 60% strain (Figure 3G). These results suggest that the multiple GTO layers and *m*Gr‐TiO_2−_ *_x_* interfaces share and effectively disperse the mechanical stress. Thus, the superior stretchability of the *n*GTO/PDMS, along with its excellent conductivity, is conserved under the harsh mechanically stressed conditions.

Because transferred graphene is rich in defects, its FET mobility increases in direct proportion to the channel length.^[^ ^21^, ^41^, ^42^ ^]^ Moreover, a decrease in the mobility due to the defect‐induced transition from the diffusive to the quasi‐ballistic transport mode has been observed even in single‐crystalline graphene grown on single‐crystalline Cu (111).^[^ ^21^ ^]^ However, the critical dimensional regulation of graphene‐FET channel length interferes with the high‐density integration of the graphene‐based devices, thus diluting the practical value of graphene.

In the present work, a stretchable bottom‐gate‐FET device with gate, active layer, and source/drain electrodes was constructed using the GTO grown at 100 °C on a stretchable PDMS (200 µm) substrate with a polyimide (120 nm) gate dielectric. Fully imidized aromatic polyimide is well known as an organic gate dielectric for flexible FETs because of its excellent chemical and electrical stability as well as flexibility compared with brittle inorganic insulators such as SiO_2_ and Al_2_O_3_.^[^ ^43^, ^44^ ^]^ In the present work, the channel length of the GTO‐FETs was varied between 10 and 100 µm at a fixed channel width of 20 µm, especially, the defect‐free nature of the GTO made it possible to decrease the channel length to 10 µm. Considering the inherent interface‐related contact issues in FETs, a gated‐transfer line method was applied to appropriately estimate the mobility as a dependency of the channel resistance to the channel length (Figure S12, Tables S1 and S2, Supporting Information^[^ ^45^ ^]^). For the GTO‐FET with a 20 µm × 20 µm channel (length × width), the drain current–gate voltage and gate current–gate voltage profiles in **Figure** **4**A reveal a high drain current on the order of mA and a low gate current at *V*_GS_ = ± 10 V. Meanwhile, the output characteristics shown in Figure 4B reveal a linear relationship between the drain voltage and drain current at various gate voltages. Regardless of the channel length, the averaged mobility of 42 GTO‐FET devices plotted in Figure 4C indicates high hole and electron mobilities of 21 000 and 12 000 cm^2^ V^−1^ s^−1^, respectively, at a *V*_GS_ = ± 4 V. Notably, this hole mobility higher than the previously reported values of 11 000 and 3000 cm^2^ V^−1^ s^−1^ for fold‐free transferred single‐crystal graphene with channel lengths of 100 and 8 µm, respectively.^[^ ^21^ ^]^ Moreover, the consistent high mobility, which does not vary with the channel length, confirms that the GTO‐FETs can be directly applied to nanometer‐scale graphene‐FETs, thus overcoming the defect density‐related channel length regulation issue. As shown in Figure 4D, the hole mobility of the GTO‐FET is maintained above 80% of the initial value even under the extreme condition of 140% strain parallel or perpendicular to the current direction. Furthermore, Figure 4E demonstrates that the inherent high mobility of the GTO‐FET is conserved during 5000 cycles of parallel stretching of up to 140% strain. Finally, the LED switching test results in Figure 4F and Video S2 in the Supporting Information demonstrate the excellent electronic reliability of the GTO‐FETs under mechanical stress. Here, the LED light intensity displays only slight decreases of ≈9.4% and ≈3.7% at 140% parallel and perpendicular strains, respectively.

**Figure 4:**
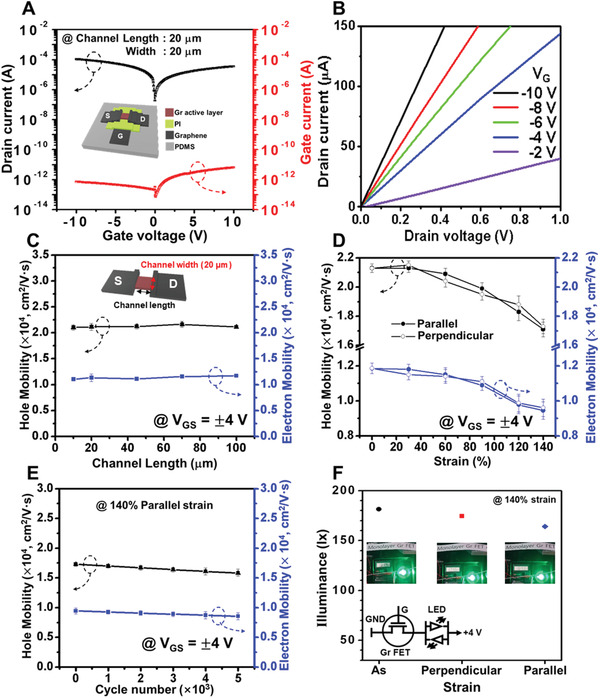
Ultra‐high mobility and flexibility of the GTO‐FETs. A) Transfer characteristics (drain current vs gate voltage and gate current vs gate voltage) of the GTO‐FET with a 20 µm × 20 µm (length × width) channel shown schematically in the inset. The sample size is 2 × 2 cm^2^. B) Output characteristics of the GTO‐FET measured at drain voltages of 0.0–1.0 V under various gate voltages (−10 to −2 V). C) Plots of hole mobility and electron mobility against channel length (*V*_GS_ = ± 4 V, channel width = 20 µm). The error ranges in data were determined using 15 devices in each channel length. D) Plots of hole mobility and electron mobility against parallel or perpendicular stretching strain of up to 140% at *V*_GS_ = ± 4 V. E) Plots of hole mobility and electron mobility of the GTO‐FETs during 5 × 10^3^ repeat cycles of stretching up to 140% parallel strain. F) LED illuminance of the GTO‐FET when relaxed (black dot) and when stretched to 140% parallel (blue diamond) or perpendicular (red square) to the current direction.

In summary, the in situ PATCVD method was implemented to directly synthesize *m*Gr on Ti‐buffered stretchable substrates without the need for a transfer step. The resulting GTO and *n*GTOs revealed ground‐breaking mechanical performance coupled with consistent and excellent electronic properties. These properties were confirmed by the fully stretchable GTO‐FET, which displayed high and consistent mobility irrespective of the channel length. The methodological advance achieved by the use of PATCVD for the low‐temperature growth of large‐area, high‐quality graphene provides a technological breakthrough toward the realization of graphene‐based soft devices and next‐generation nanometer‐scale FETs.

## Conflict of Interest

The authors declare no conflict of interest.

## Author Contributions

Y.H., B.‐J.P., and J.‐H.E. contributed equally to this work. S.G.Y. planned and supervised the project; Y.H., B.J.P., J.H.E., and S.G.Y. designed and performed the experiments, and characterized the electrical properties of graphene; V.J., S.I., and S.V.N.P. fabricated the PDMS substrate and designed the LED circuit; J.S.C. analyzed the crystallinity and phase of the graphene; H.H., H.C., and H.Y.K. performed DFT calculations; C.J. analyzed in situ deposited Ti metal via XPS; K.P., H.T.J., S.Y., Y.H.K. analyzed the grain size of the graphene using liquid crystal films; H.Y.K., Y.H.K., S.G.Y. analyzed the results and wrote the manuscript.

## Supporting information

Supporting Information

Supplemental Video 1

Supplemental Video 2
