# Supplementary material for: Direct Growth of Highly Conductive Large‐Area Stretchable Graphene
Source: Adv Sci (Weinh). 2021 Feb 1;8(7):2003697. doi: 10.1002/advs.202003697 (PMC8025006; doi:10.1002/advs.202003697)
Supplement: Supplementary file 1 — Supporting Information [file ADVS-8-2003697-s002.pdf]

© 2021 Wiley-VCH GmbH

ADVANCED SCIENCE
Open Access

Supporting Information

for *Adv. Sci.,* DOI: 10.1002/advs.202003697

Direct Growth of Highly Conductive Large-Area Stretchable Graphene

Yire Han, Byeong-Ju Park, Ji-Ho Eom, Venkatraju Jella, Swathi Ippili, S. V. N. Pammi, Jin-Seok Choi, Hyunwoo Ha, Hyuk Choi, Cheolho Jeon, Kangho Park, Hee-Tae Jung, Sungmi Yoo, Hyun You Kim, Yun Ho Kim,* and Soon-Gil Yoon**

Supporting Information

Direct Growth of Highly Conductive Large-Area Stretchable Graphene

Yire Han,[1ξ] Byeong-Ju Park,[2ξ] Ji-Ho Eom,[1ξ] Venkatraju Jella,[1] Swathi Ippili,[1] S. V. N. Pammi,[1] Jin-Seok Choi,[3] Hyunwoo Ha,[1] Hyuk Choi,[1] Cheolho Jeon,[4] Kangho Park,[5] Hee-Tae Jung,[5] Sungmi Yoo,[6] Hyun You Kim,[1*] Yun Ho Kim,[6,7*] and Soon-Gil Yoon[1*]

[1]Department of Materials Science and Engineering, Chungnam National University, Daeduk Science Town, 34134, Daejeon, Republic of Korea

[2] P&T Division, SK Hynix, Cheongju, 28433, Republic of Korea

[3]Analysis Center for Research Advancement (KARA), Korea Advanced Institute of Science and Technology, 291 Daehak-ro, Yuseong-gu, 34141, Daejeon, Republic of Korea

[4]Advanced Nano-Surface Group, Korea Basic Science Institute (KBSI) 169-148 Gwahangno, Yuseong-gu, Daejeon 34133, Republic of Korea

[5]Department of Chemical and Biomolecular Engineering, Korea Advanced Institute of Science and Technology, Daejeon 34141, Republic of Korea

[6]Advanced Materials Division, Korea Research Institute of Chemical Technology, Daejeon, 34114, Republic of Korea

[7] Department of Chemical Convergence Materials and Processes, KRICT School, University of Science and Technology, Daejeon, 34114, Republic of Korea

*Corresponding authors: sgyoon@cnu.ac.kr, yunho@krict.re.kr, hykim@cnu.ac.kr

[ξ]These authors are equally contributed to this work.

CONTENTS

1. Supporting Tables and Figures

2. Materials and methods

3. References

4. Videos

1. Supporting Tables and Figures

Table 1 Dependency of R_{ch} (hole transport) on channel length at a channel width of 20 μm at V_{GS} = - 4V.

Channel Length (μm)	10	20	45	70	100
R_{TOT} (Ω)	529	996	2,176	3,386	4,686
R_c (Ω)	55	55	55	55	55
R_{ch} (Ω)	474	941	2,121	3,331	4,631

Table 2 Dependency of R_{ch} (electron transport) on channel length at V_{GS} = + 4V.

Channel Length (μm)	10	20	45	70	100
R_{TOT} (Ω)	701	1,315	2,927	4,354	6,132
R_c (Ω)	157	157	157	157	157
R_{ch} (Ω)	544	1,158	2,770	4,197	5,975

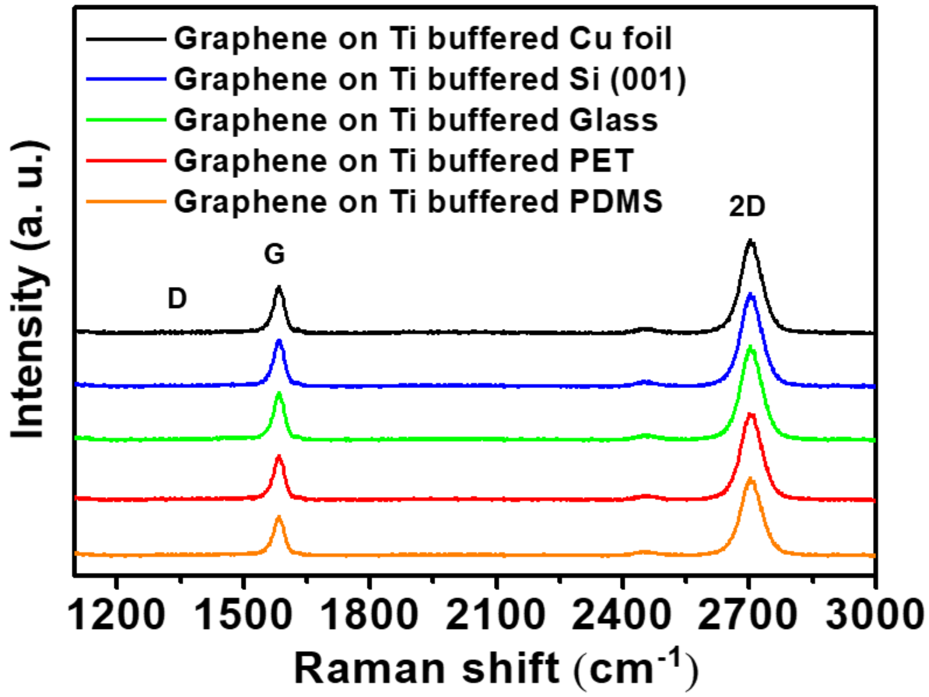

Fig. 1 Raman spectra of monolayer graphene grown on Ti (10 nm)-buffered PDMS, PET, Glass, Si (001), and Cu foil substrates at 100 °C.

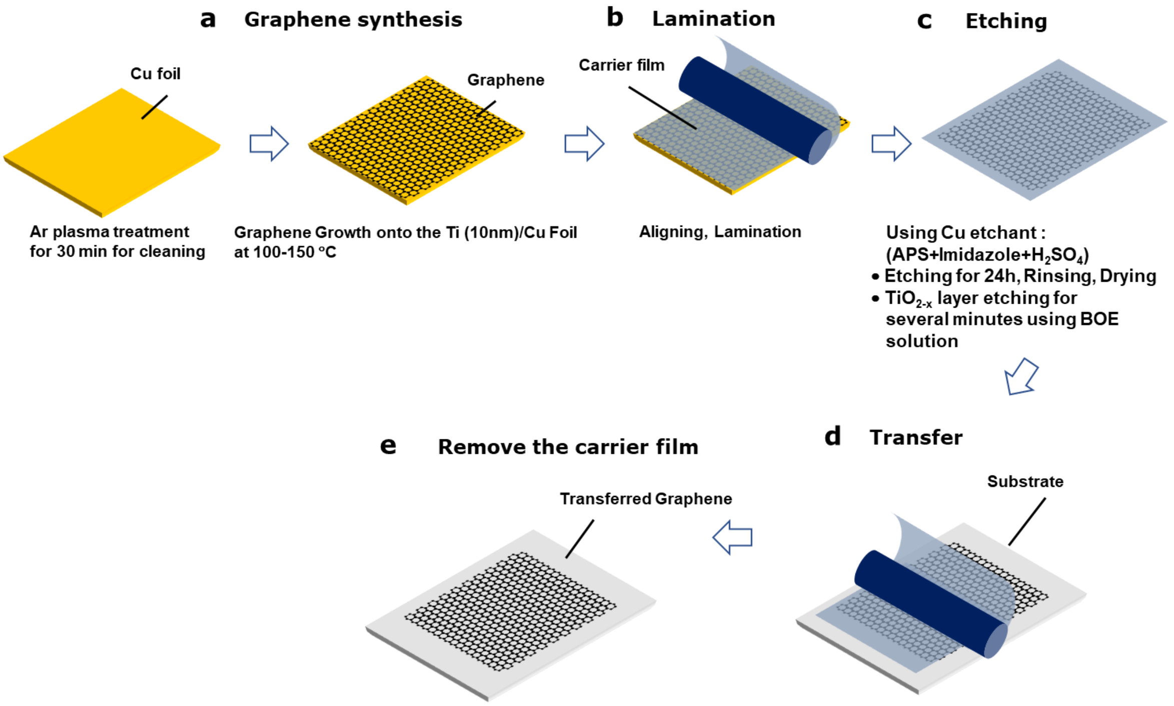

Fig. 2 Dry-transfer process without surface modifications of the graphene grown directly onto the Ti-buffered Cu foil. (**a**) Graphene growth onto the Ar-plasma treated Cu foil at 100-150 °C. (**b**) The TRT film as a carrier film that is widely used for transferring graphene was attached *via* a roller onto the graphene/TiO_{2-x}-buffered Cu foil sample. (**c**) TRT/Graphene/TiO_{2-x}/Cu foil was dipped for 24h using etching solution of Cu foil, and after Cu foil etching, the etched samples were again etched for several minutes using BOE (a 6:1 volume ratio of 40% NH_4F in water to 49% HF in water) solution for residual TiO_{2-x} etching. (**d**) The graphene side of graphene/carrier film was attached onto the transferred substrate and the roller was rolled on the carrier film at 110 °C in order to detach the carrier film because the carrier film loses adhesion with the graphene at temperatures equal to or exceeding 90 °C. (**e**) Finally, graphene was transferred to the various substrates.

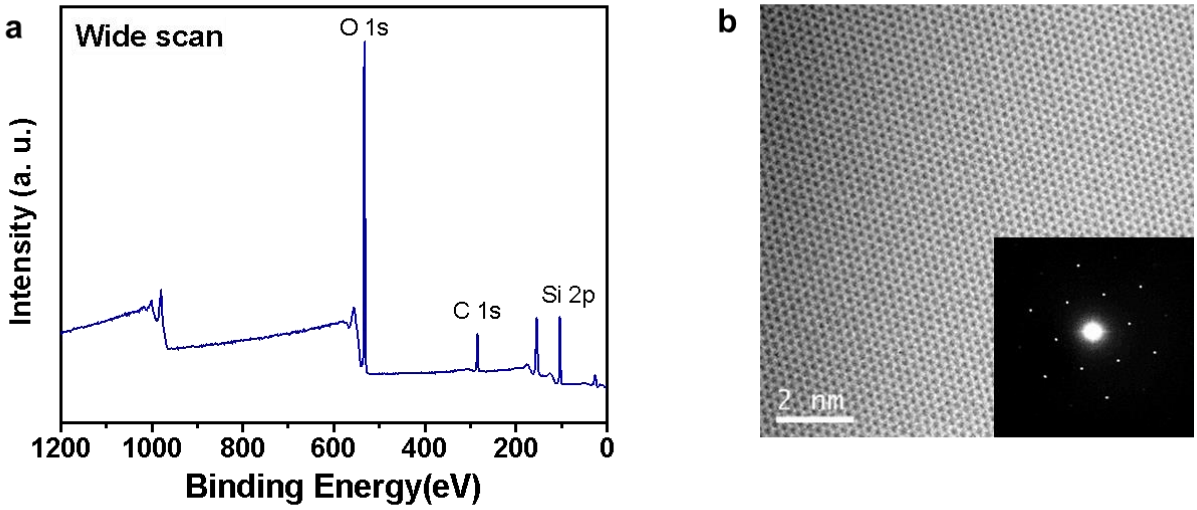

Fig. 3 (a) XPS wide scan (measurement area of one point: 300 μm) of the graphene transferred to the SiO_2/Si substrate *via* a dry transfer process. Here, XPS spectra showed no Ti and TiO_{2-x} phases at 4-point measurements for a 1 × 1 cm^2 graphene area. **(b)** HRTEM image and selected-area electron diffraction (SAED) pattern (inset).

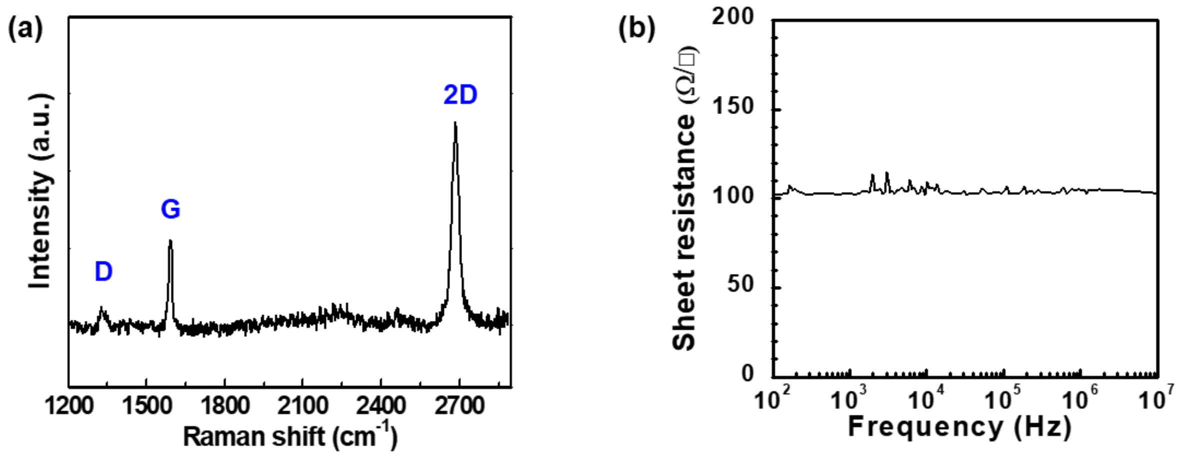

Fig. 4 (a) Raman spectrum of transferred graphene without TiO_{2-x} layer and (b) Sheet resistance of transferred graphene measured *via* Z-theta method.

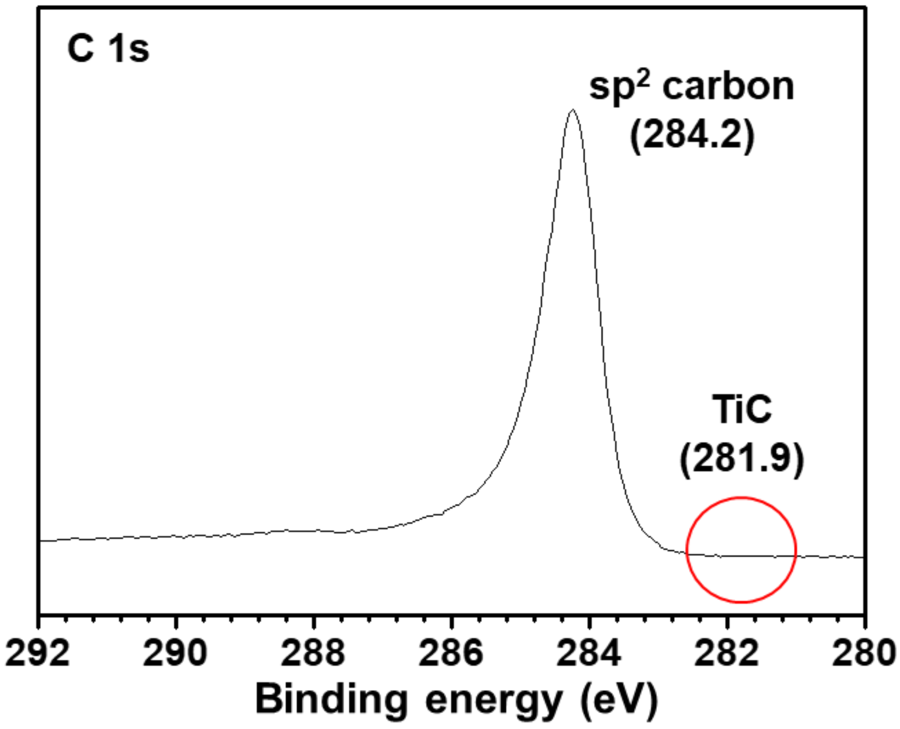

Fig. 5 XPS C1s core level exhibiting no formation of titanium carbide (281.9 eV) formed by the reaction between carbon (graphene) and a Ti-buffer layer at 100 °C (red circle). The existence of C1s at 284.2 eV refers to the sp^2 graphene.

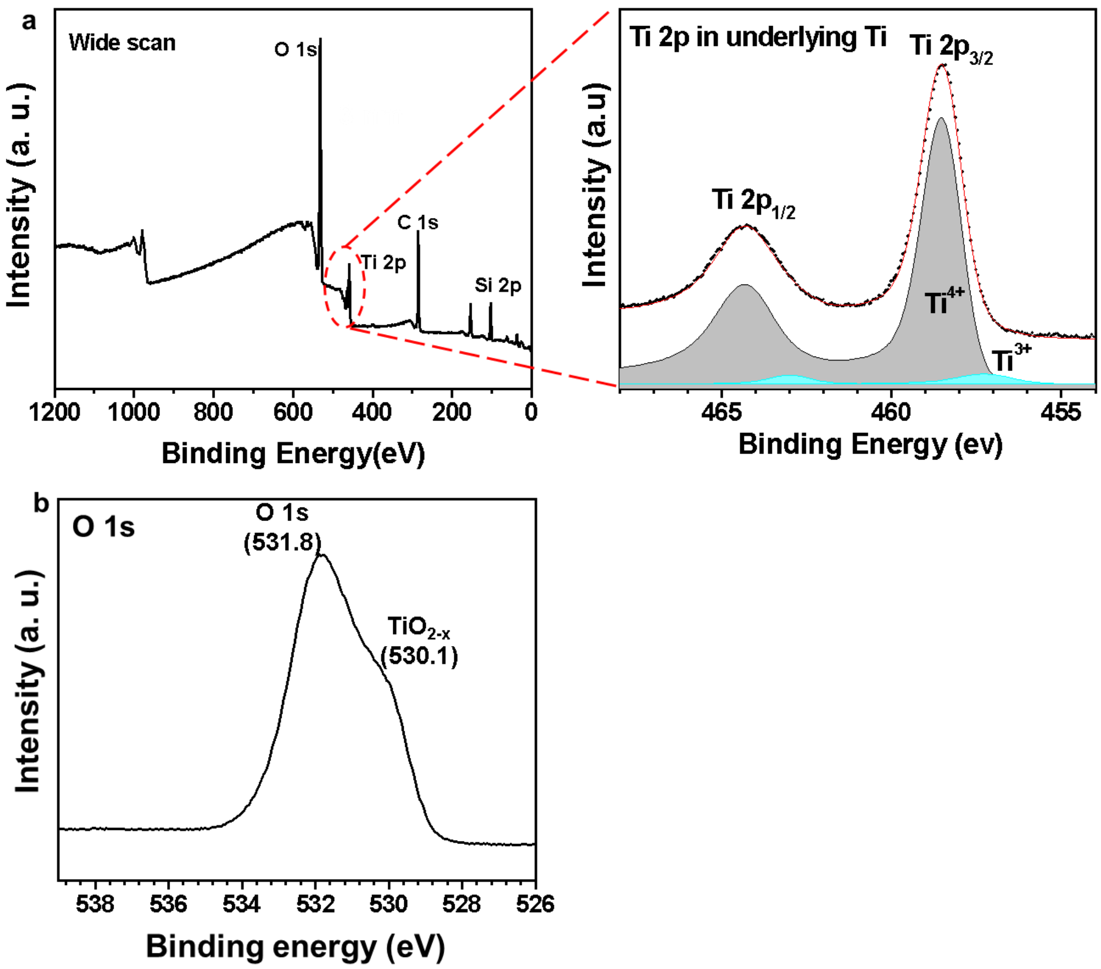

Fig. 6 (a) XPS wide scan of monolayer graphene that was exposed under ambient conditions after a growth of graphene on a Ti (10 nm)-buffered Si (001) substrate at 100 °C. Here, Si 2p peaks were observed from a Si substrate, which means the analysis of full layer of graphene/TiO_{2-x}(10nm) grown on SiO_2/Si substrate. The XPS enlarged spectrum of a Ti 2p core level (right figure) is indicated by a dotted circle from an XPS wide scan (**a**). The oxidation states of Ti are composed of Ti^{+4} and Ti^{+3} with no Ti metal layer. **(b)** XPS O1s core level in graphene/TiO_{2-x} (10 nm). Here, an oxygen under ambient conditions oxidized the Ti-layer to TiO_{2-x} as well as an adsorption onto the graphene films (O1s).

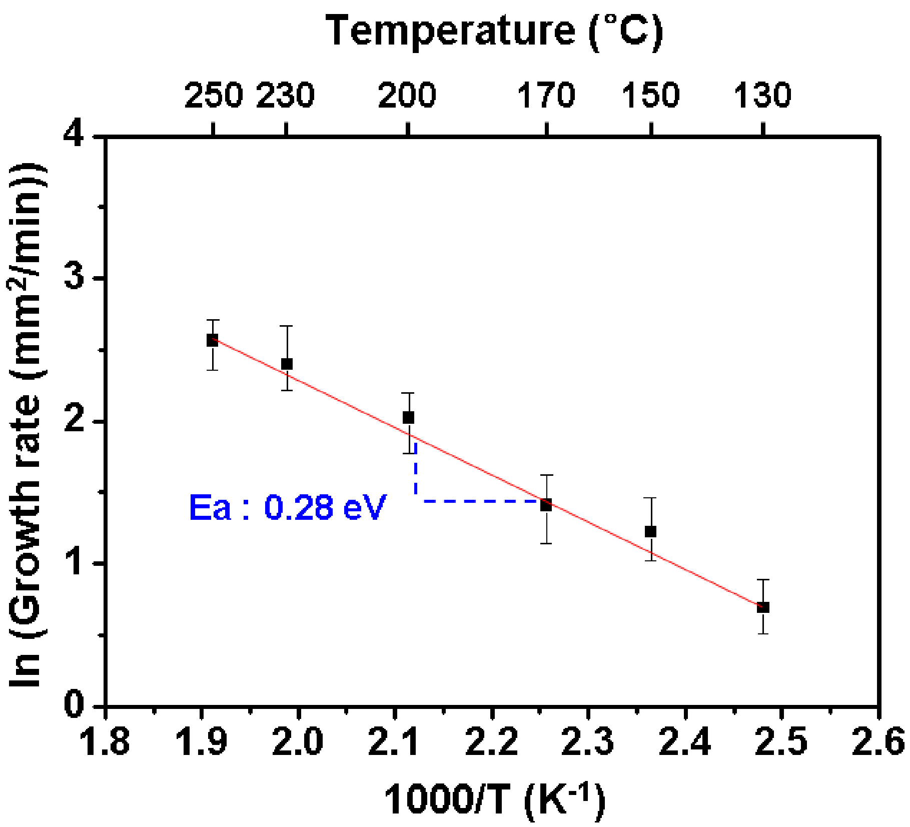

Fig. 7 Arrhenius plot of the graphene growth rate at different temperatures for a mechanism of graphene growth.

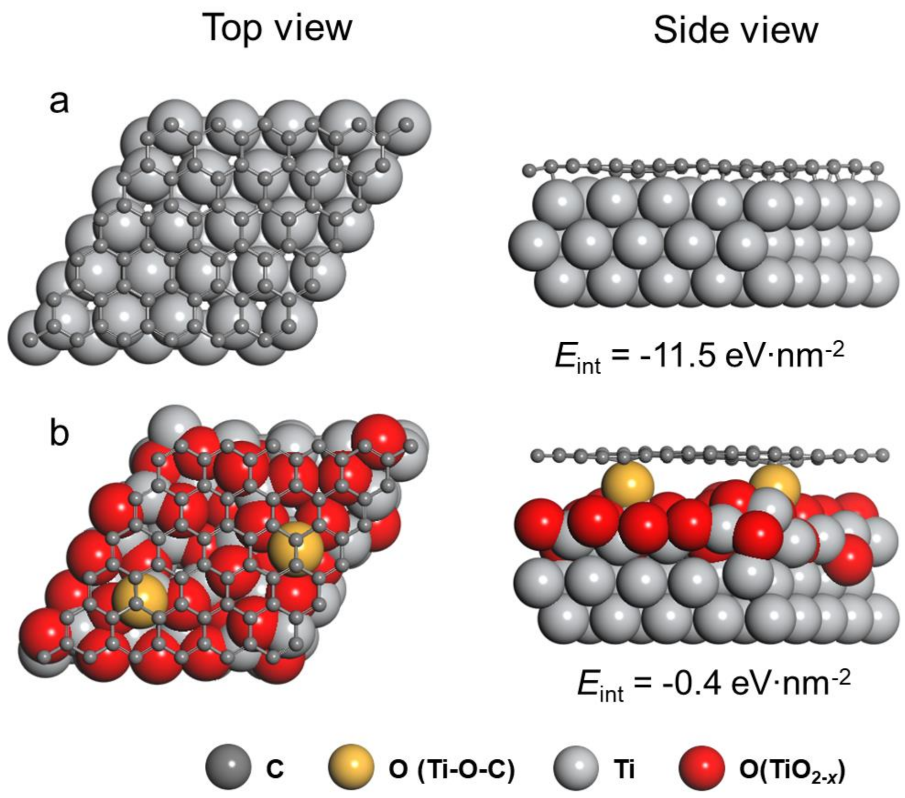

Fig. 8 DFT-optimized monolayer graphene supported on **(a)** Ti and **(b)** TiO_{2-x}. The E_{int} presents the area-normalized interaction energy between a monolayer graphene and Ti (or TiO_{2-x}).

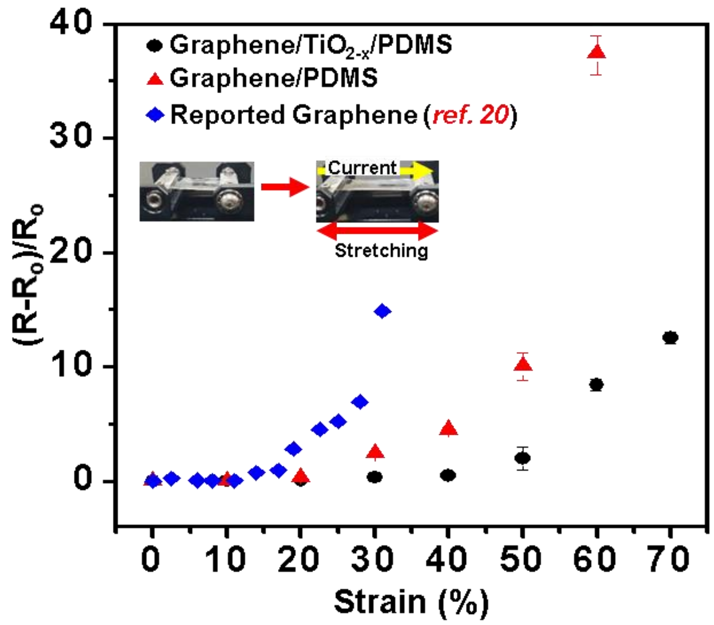

Fig. 9 Normalized resistance change of graphene/TiO_{2-x}/PDMS and graphene/PDMS (without TiO_{2-x} layer by transfer) under parallel strain to the direction of current flow. Reported graphene means the transferred graphene grown onto the Cu foil at 1,000 °C.

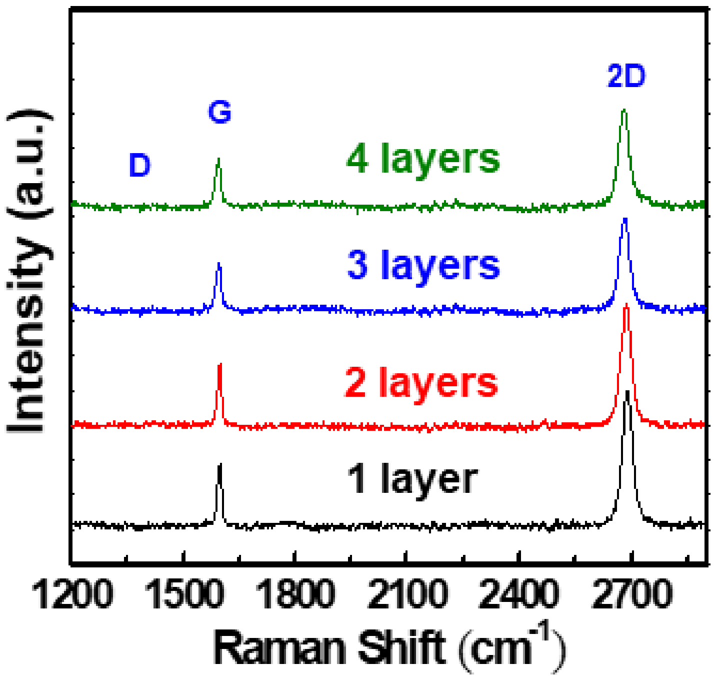

Fig. 10 Raman spectrum of each stacking individual layer in multilayered GTOs.

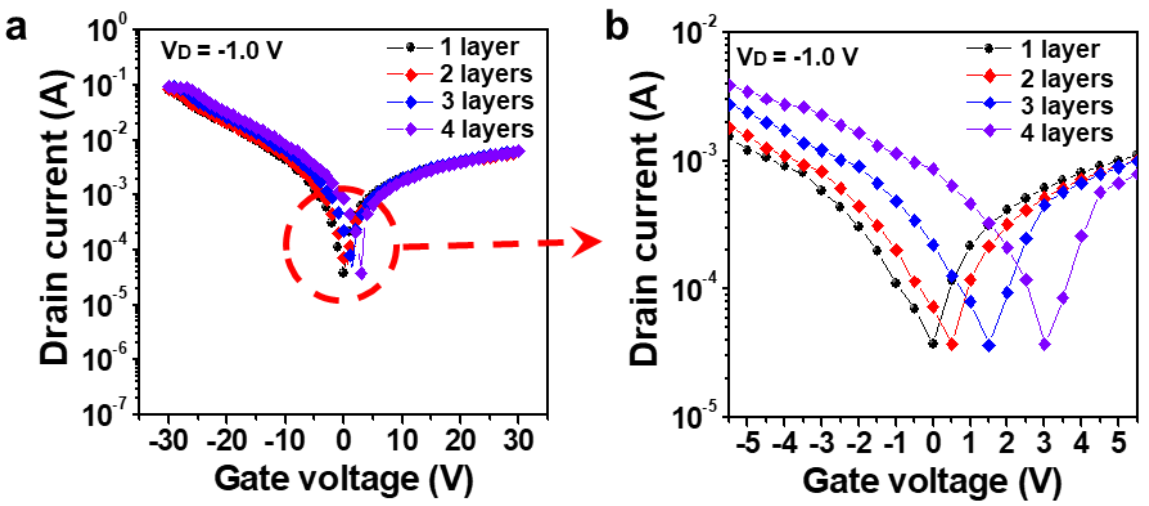

Fig. 11 **(a)** Transfer characteristics of multi-stacked *n*GTO-FETs measured at ambient conditions after annealing for 2h at 250 °C under high vacuum (1.3×10^{-4} Pa) and **(b)** Enlarged version of the small gate voltage from **(a)**. Here, channel width and length of FETs are 800 and 200 μm using a shadow mask, respectively.

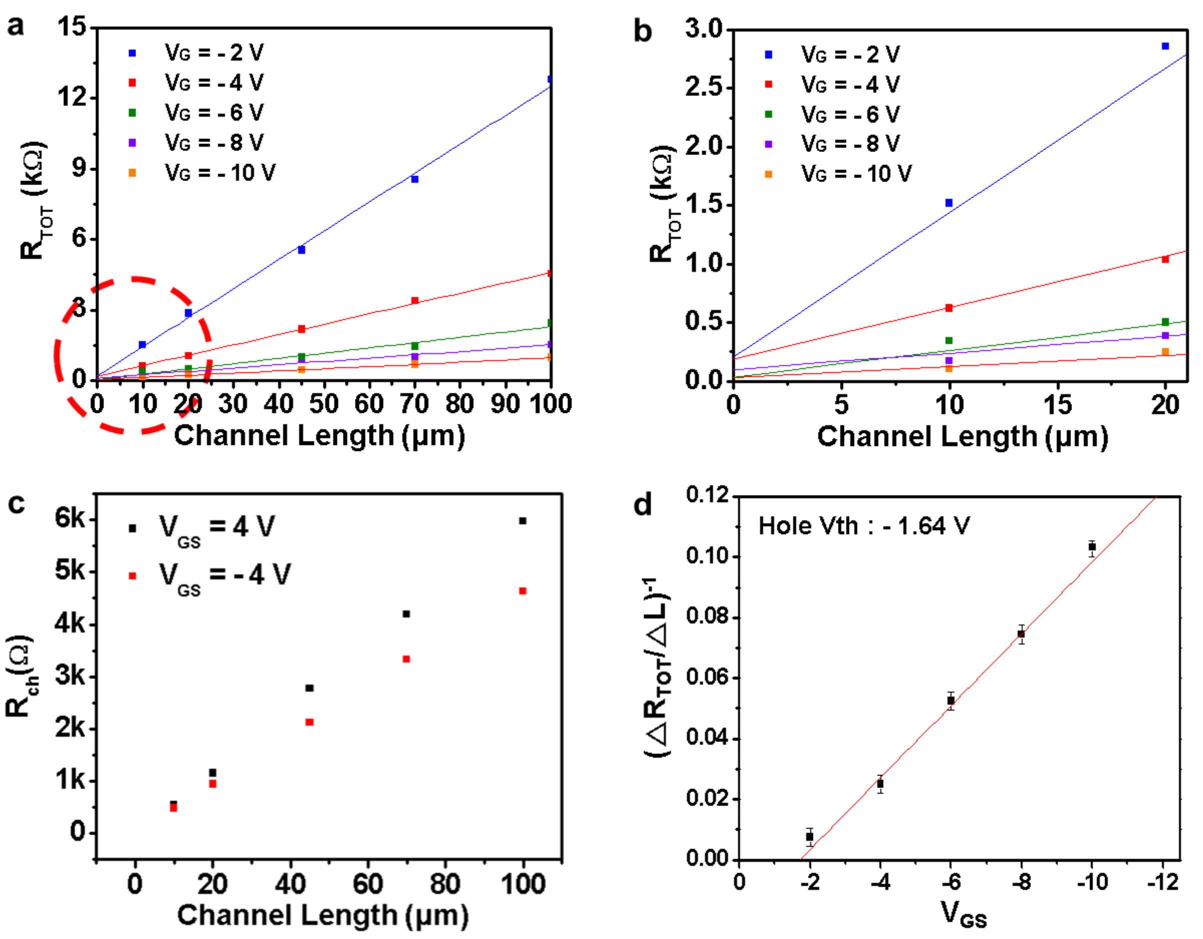

Fig. 12 **(a)** Relationship between R_{TOT} and channel length for different gate voltages at a channel width of 20 μm. **(b)** Enlarged version from the dotted red circle of **(a)** for determination of R_C. **(c)** Channel resistance of holes and electrons with a linear relationship for different channel lengths. **(d)** Determination of hole threshold voltage (V_{TH}) from the relationship between $(\Delta R_{TOT}/\Delta L)^{-1}$ and gate voltage. The V_{TH} of hole was -1.64 V.

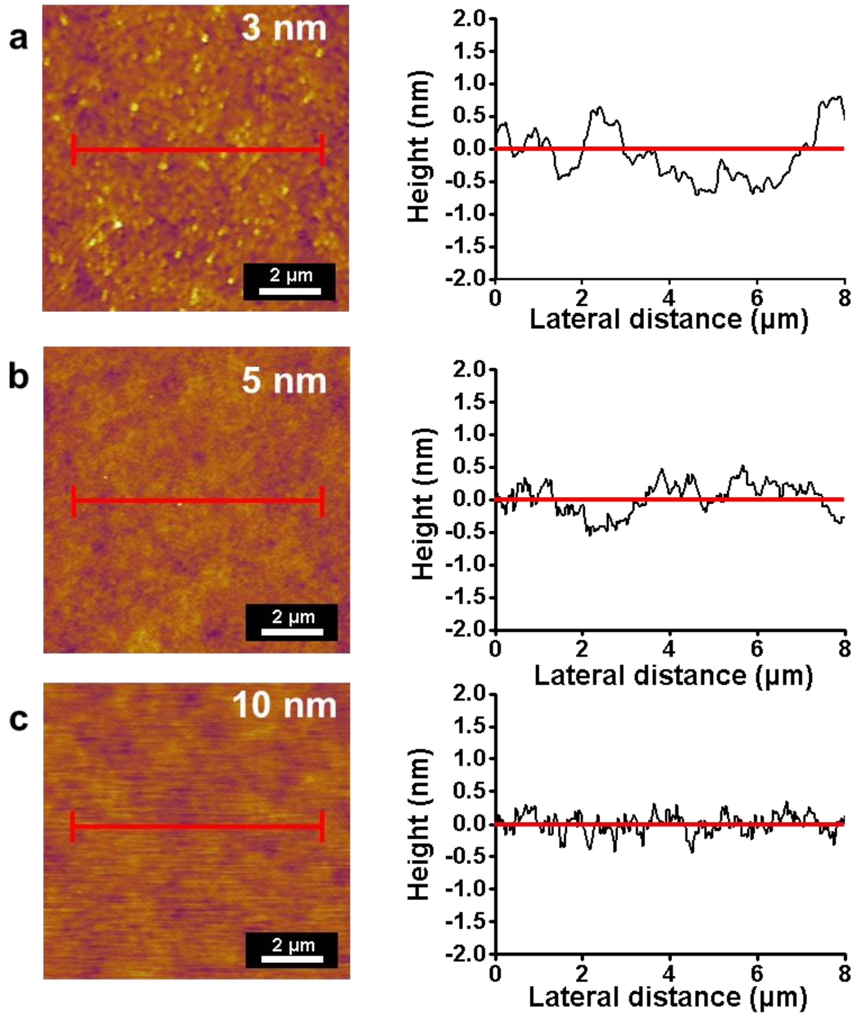

Fig. 13 AFM surface images of **(a)** 3 nm, **(b)** 5 nm, and **(c)** 10 nm-thick TiO_{2-x} layers on PET substrates. Step heights of the 3 nm, 5 nm, and 10 nm-thick TiO_{2-x} layers measured along the lateral distance (indicated by the solid red line) of the AFM images are presented together. The 10 nm-thick TiO_{2-x} layers showed a step height of approximately ± 0.2 nm, indicating a homogeneous surface morphology. The rms roughness of the 3 nm, 5 nm, and 10 nm thick TiO_{2-x} layers is 0.22 ± 0.05 nm, 0.19 ± 0.03, and 0.17 ± 0.02 nm, respectively.

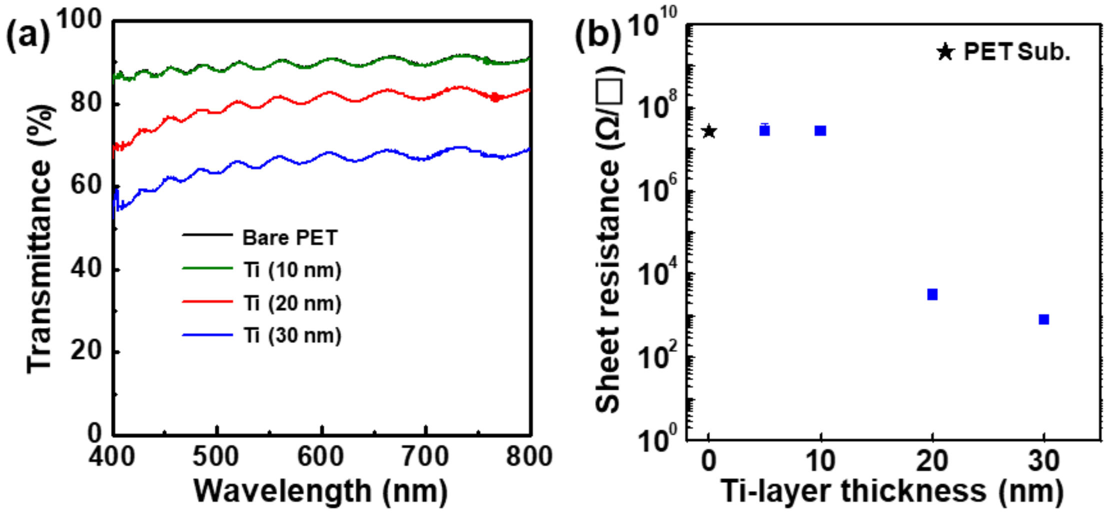

Fig. 14 (a) Transmittance and (b) sheet resistance of various thicknesses of Ti-buffer layers deposited onto a PET substrate. The Ti-layers below 10 nm thickness did not vary the transparency and the resistance of the substrates.

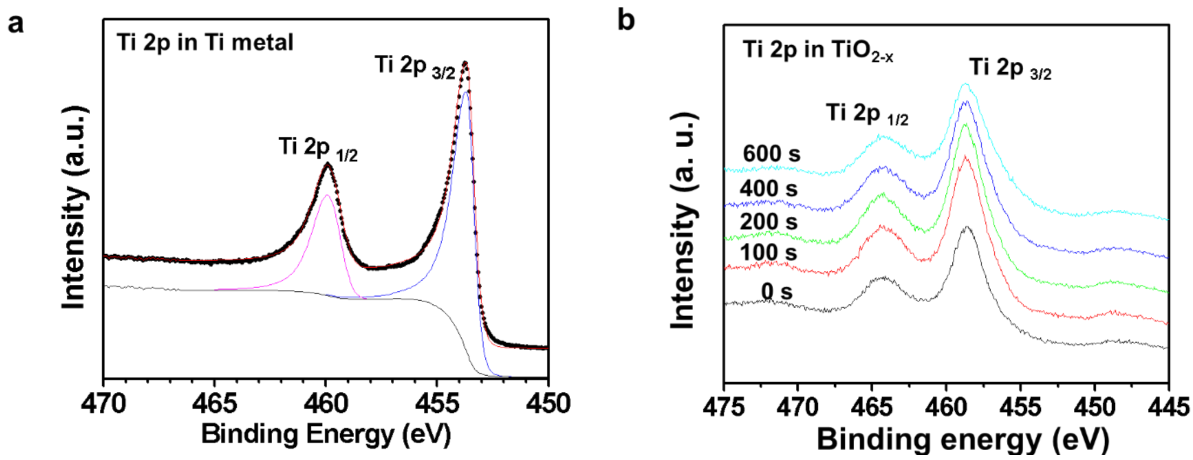

Fig. 15 XPS spectra of a Ti-buffer layer and an oxidized TiO_{2-x} layer. (a) XPS Ti 2p spectrum recorded without breaking the vacuum after deposition of a Ti (10 nm)-buffer layer. **(b)** XPS depth-profile of the TiO_{2-x} (10 nm)-buffer layer etched for 0 to 600s after exposure under ambient conditions.

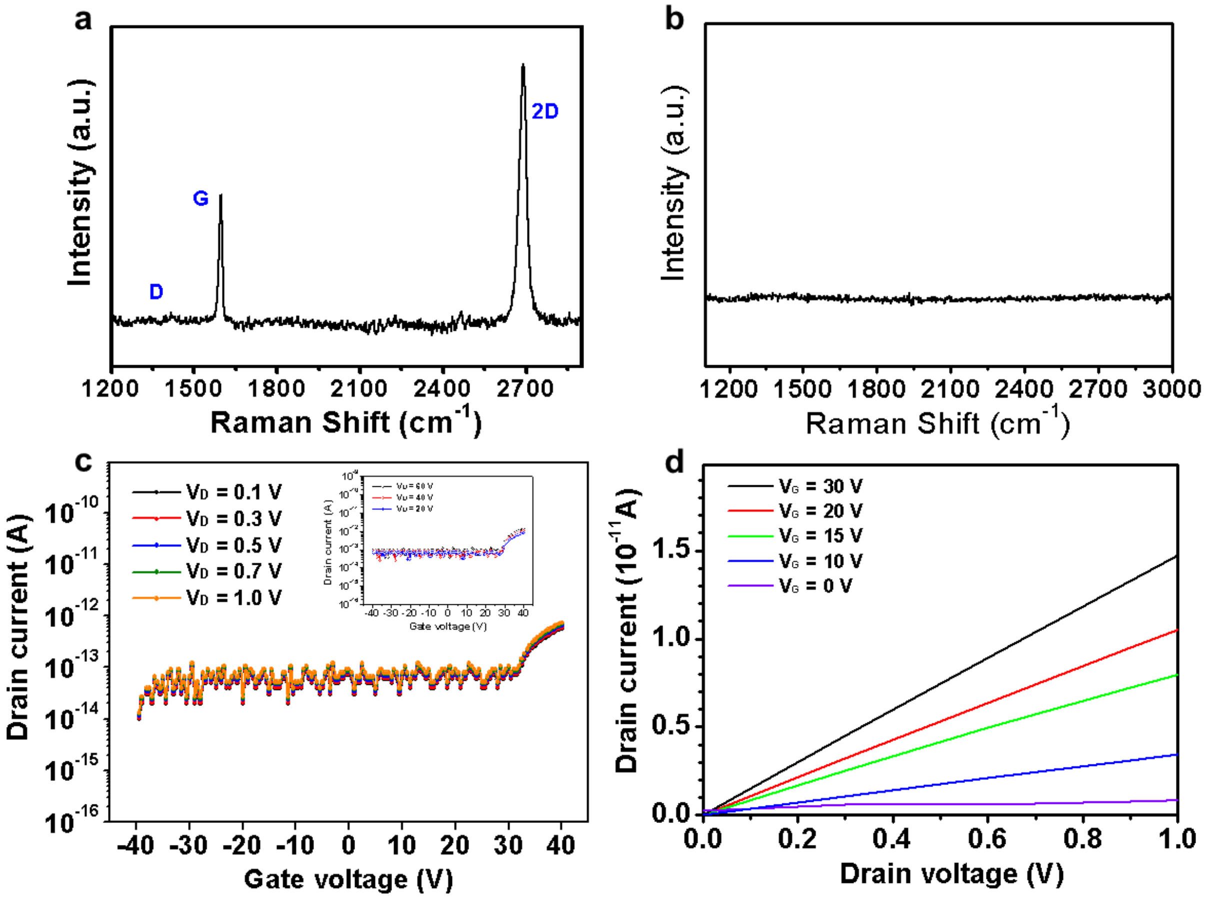

Fig. 16 Raman spectra of (**a**) GTO/SiO_2 (300 nm)/highly-doped p-type Si (001) substrate and of (**b**) graphene etched completely *via* argon plasma. (**c**) and (**d**) Transfer (at various V_D) and output characteristics (at various V_G) observed at 10 nm-thick TiO_{2-x}-buffered FET device without graphene, respectively. Inset in (**c**) showed transfer characteristics observed at higher drain voltages (V_D).

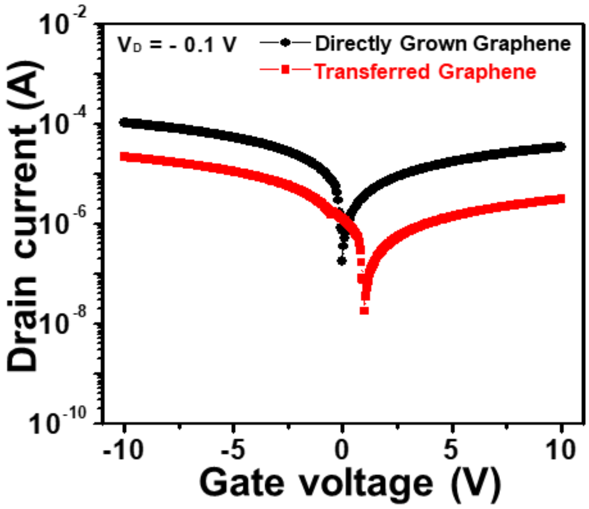

Fig. 17 Transfer characteristics of the graphene-FETs. Transferred graphene-FETs were fabricated using a graphene active layer transferred to the SiO_2 (300 nm)/highly-doped p-type Si (001) substrate. Source/Drain electrodes are GTO. Transferred graphene-FETs showed a positive shift of Dirac voltage to 1.0V. Here, channel width and length are 20 and 20 μm, respectively, using a photolithography process.

2. Materials and methods

2.1 Deposition of Ti on PET and PDMS substrates, and subsequent analysis

The 10 nm-thick Ti films were deposited at 100 °C on PET (100 μm) and PDMS (200 μm) substrates *via* dc sputtering under the following conditions: dc power of 25W, base pressure of 6.6×10^{-4} Pa, working pressure of 0.4 Pa, deposition time of 10 min, and distance between target and substrate of 10 cm. A mixture of argon and hydrogen was used during sputtering for the deposition of a clean Ti layer. The thickness of Ti-buffer layer was conveniently controlled by deposition parameters such as dc power, working pressure, deposition time, and the ratio of Ar/H_2. The optimal thicknesses of Ti-buffer layer were analyzed *via* TEM cross-sectional image, AFM image using the patterned steps, and Ellipsometer laser measurement. The smoothness of the surface with different Ti thicknesses was determined based on atomic force microscopy (AFM) analysis of the oxidized TiO_{2-x} surface (see main text and Supporting Fig. 13). As shown in Supporting Fig. 14, the Ti-layers below 10 nm thickness did not vary the transparency and the resistance of the substrates because the Ti-layers were naturally oxidized as a TiO_{2-x} under ambient air at room temperature (XPS depth-profile of Supporting Fig. 15(b)). Therefore, in this study, thickness of the Ti-buffer layer was determined at 10 nm for graphene growth.

The electronic state of Ti-buffer layer deposited under an argon and hydrogen atmosphere *via* dc sputtering was *in situ* analyzed *via* XPS without breaking the vacuum. XPS spectra presented in Supporting Fig. 15a revealed typical spectra of metallic Ti. To remove the oxygen that may exist in a reactor under a base pressure of 1.3×10^{-4} Pa, hydrogen was flowed into a reactor during the Ti deposition. The XPS spectra of the naturally oxidized Ti under ambient conditions shows a typical feature of TiO_{2-x} (Supporting Fig. 15b).

The thickness of the Ti layer was confirmed *via* transmission electron microscopy (TEM)

and AFM cross-sectional imaging. Sheet resistance and transmittance were measured using a Z-theta method and a UV-vis spectrometer, respectively. The rms roughness of Ti films annealed at different temperatures under a hydrogen atmosphere was measured *via* AFM (MFP-3D-BIO, Asylum Research) at room temperature to examine their thermal stability. The Ti before and after graphene growth was investigated *via* X-ray photoelectron spectroscopy (XPS).

2.2 Large-area graphene synthesis at low temperatures via PATCVD on Ti-buffered substrates and subsequent analysis

Large-area *m*Gr (4-in-wafer) was synthesized at low temperatures *via* PATCVD. After depositing Ti, PATCVD was performed for *m*Gr growth under the following optimal conditions: rf power of 70 W, base pressure of 1.3×10^{-4} Pa, working pressure of 2.4×10^{2} Pa, growth time of 2h, a gas flow rate of $Ar/H_2/CH_4$: 10/25/3.0 sccm (standard cc min^{-1}), a distance between the rf sources of 8 cm, and a distance of 10 cm between the rf sources and a substrate (see Fig. 1A). The growth temperature was controlled under 150 °C.

The electronic state of the 10-nm-thick Ti films synthesized *via* dc sputtering was analyzed *via* XPS without breaking the vacuum. The XPS spectra was obtained using an AXIS Ultra-DLD (Kratos Inc.) with a monochromatic Al Kα X-ray (hν = 1486.6 eV), which was located at the Korea Basic Science Institute (KBSI). The XPS analyzer was connected to a high-vacuum dc/rf (radio-frequency) sputter system *via* an ultra-high vacuum chamber, which allowed us to measure the Ti 2p spectrum of Ti thin films without exposing the sample to air. After subtraction of the Shirley background, the Ti 2p spectrum was fitted using Doniach-Sunjic functions with an asymmetric line-shape.[46]

For annular dark field (ADF)-TEM analysis of *m*Gr, a 200-nm-thick SiO_2 layer was deposited on *m*Gr that was free of plasma damage *via* facing-target sputtering before the sample preparation *via* focused ion beam (FIB). The crystallinity of the graphene was determined *via*

high-resolution TEM (HRTEM) and the selected area electron diffraction (SAED) patterns of the *m*Gr were acquired from the *m*Gr transferred to a copper grid using a damage-mitigated dry transfer process (Supporting Fig. 2).[47] In order to exclude the effect of the 10 nm-thick TiO_{2-x} layer on the sheet resistance and mobility of the graphene, graphene grown directly onto the 10 nm-thick Ti-buffered Cu foil was transferred to the SiO_2(100nm)/Si substrate after etching for 24h. The complete etching of the TiO_{2-x} layer was performed for several minutes using a BOE (NH_4F: HF = 6:1) solution after Cu foil etching for 24h.

The transmittance of monolayer graphene was measured *via* UV-vis spectrometer. The sheet resistance of the graphene was measured *via* the Z-theta method using an impedance/gain-phase analyzer (HP4194A) in a range of 100 Hz-10 MHz.

To estimate the domain size of *m*Gr using 5CB (4-pentyl-4'-cyanobiphenyl), liquid- crystal films (< 2 μm) were spin-coated onto the graphene films at 500-3,000 rpm. The textures of the liquid-crystals oriented on the graphene film were observed using a polarized optical microscope (POM, LV 100POL, Nikon) equipped with a 1λ wave plate and a charge-coupled device (CCD) camera.

2.3 Structural analysis of mGr

A *m*Gr was transferred to a SiO_2/Si substrate (for XPS analysis) and a Cu grid (for HRTEM analysis) to investigate a high-quality *m*Gr lattice without an underlying TiO_{2-x} layer. The transfer of *m*Gr grown at 100 °C on a Ti-buffered Cu foil was performed for 24h *via* dry etching process using a carrier film (sequential etching process of Supporting Fig. 2) without surface modifications, and then a residual TiO_{2-x} layer was completely removed using a buffered oxide etch (BOE) solution. An XPS wide scan (Supporting Fig. 3a) of the transferred graphene showed neither Ti nor TiO_{2-x} peaks, which revealed a pure pristine graphene after transfer. The existence of an oxygen on the transferred graphene (Supporting Fig. 3a) was attributed to an adsorption of oxygen onto the graphene under ambient air. Based on the high-resolution TEM

image (Supporting Fig. 3b) and a selected-area electron diffraction (SAED) pattern (inset of Supporting Fig. 3b) show the atomically thin, high-quality *m*Gr lattice.

2.4 Graphene-FET synthesis

The graphene bottom-gated-FET devices were prepared with different channel lengths from 10 to 100 μm at a channel width of 20 μm *via* a photolithography process. A 120 nm-thick polyimide gate insulator was deposited onto the gate electrode (graphene/TiO_{2-x})/PDMS *via* spin coating. The polyimide insulators used in this study were synthesized using hexafluoroisopropylidenediphthalic anhydride (6FDA) and 4,4-methylenedibenzenamine (MDA) monomers in *m*-cresol solvent, as described in our previous reports.[43,44] The source/drain graphene electrode and graphene active layer were *in-situ* grown onto the gate insulator (PI).

2.5 Mobility analysis

A *m*Gr active layer was grown on Ti (10 nm)-buffered PI (120 nm) gate insulator at 100 °C. The structure of the bottom-gated FETs is as follows: Source/Drain electrode (GTO)/graphene active layer (GTO)/gate insulator (PI)/gate electrode (GTO)/PDMS substrate. Because the contact resistance may influence the mobility, we used a gated-transfer line method (gTLM) for FET-mobility calculation, which showed a relationship between channel length and channel resistance.

$$R_{ch} \text{ (channel resistance)} = L/[\mu C_{ox} W(V_{GS}\text{-}V_{TH})] \text{ --------- (1)}$$

where, L, channel length; μ, mobility; C_{ox}, capacitance density of PI (120 nm) gate dielectric; W, channel width; V_{GS}, applied gate voltage; V_{TH}, threshold voltage.

In the linear regime (Fig. 4B), for fixed V_{GS}, R_{TOT} (total resistance) was calculated by V_{DS}/I_D, and $R_{TOT} = R_{ch}\ (L) + R_c$ (contact resistance). For determination of R_c, when the R_{TOT} was plotted against L for various V_{GS} (Supporting Fig. 12a), a straight line is obtained and it was intercepted at *y*-axis (Supporting Fig. 12b). The intercepted value is $2R_c$. The channel resistance for holes

and electrons has a direct linear relationship with the channel length, which reveals an increase in the channel resistance with increases in the channel length at V_{GS} = ± 4V (Supporting Fig. 12c). For determination of V_{TH}, the reciprocal slopes ($(\Delta R_{TOT}/\Delta L)^{-1}$) were plotted against V_{GS} and a straight line was extrapolated to *x*-axis, an intercept at *x*-axis was a value of V_{TH} (Supporting Fig. 12d). The various parameters such as R_{TOT}, R_c, and R_{CH} for different channel lengths at a channel width of 20 μm were summarized at Supporting Table 1 (for hole) and Table 2 (for electron) at a gate voltage of -4 V and +4V, respectively. We calculate the FET-mobility by an equation (1) using the values of various parameters.

To estimate the effect of underlying TiO_{2-x} to the mobility, *m*Gr (Raman spectrum, Supporting Fig. 16a) formed on TiO_{2-x} (GTO) was etched for one minute *via* an argon-plasma to completely remove *m*Gr, Supporting Fig. 16b). The FETs fabricated without a *m*Gr active layer exhibited no signal in transfer characteristics (I_{ds}-V_{gs}) (Supporting Fig. 16c) and a low-drain current at I_{ds} = 1.0 V (Supporting Fig. 16d). As a result, the underlying TiO_{2-x} layer did not influence the FET-mobility of *m*Gr. We compared with the graphene-FET characteristics using no transferred and transferred graphene active layer (Supporting Fig. 17). The transfer characteristics of the graphene-FETs with no transferred graphene active layer revealed ambipolar characteristics with a Dirac voltage of 0.0V. On the other hand, transfer characteristics with a transferred graphene active layer showed a similar shape with the exception of a shift in the Dirac voltage to the positive of 1.0V and a reduced drain current that was frequently observed in the transfer process. These results confirmed that the TiO_{2-x} layer does not influence the graphene-FET characteristics.

2.6 Stretching tests

The normalized resistance change, $(R-R_o)/R_o$, of a 70 μm × 15,000 μm GTO and *n*GTO strips grown onto a 3.0 cm × 4.0 cm PDMS substrate was presented as a function of parallel and perpendicular stretching strain to the current flow and was addressed under 10^4 cyclic strain

loading up to 60% parallel strain.

The hole and electron mobilities of *m*Gr-FETs with a micrometer-scale channel dimensions were demonstrated for parallel and perpendicular strains up to 140% and under 5,000 cycles at 140% parallel strain. Demonstration of stretchable LED control units by *m*Gr-FETs was performed at 140% parallel and perpendicular strains using the circuit of *m*Gr-FETs to drive LED.

2.7 Density functional theory calculations

The Ti(0001) lattice was modeled with a 6 × 6 × 3 slab. A 15 Å of vacuum space was secured to describe the surface. The bottom Ti layer was fixed during optimization, and a single graphene layer composed of 72 carbon atoms was optimized on the Ti(0001) lattice. The details of sequential monolayer graphene (*m*Gr) formation on Ti(0001) can be found in our previous report.[22] To describe the TiO_{2-x} substrate, total 32 oxygen atoms were added to the upper two atomic layers of the Ti slab. The interaction between *m*Gr and TiO_{2-x} was estimated by optimizing a *m*Gr layer (composed of 72 carbon atoms) on the optimized TiO_{2-x} surface.

We performed GGA (Generalized Gradient Approximation) levels of spin-polarized DFT calculations with the Vienna ab-initio simulation package (VASP) [48] and the Perdew-Burke-Ernzerhof (PBE) functional.[49] Plane waves up to an energy cutoff of 400 eV were used to describe the valance electrons. The projector augmented wave method was applied to describe the interactions between core ions and valance electrons.[50] The Brillouin zone was sampled at the Γ-point. The convergence criteria for the electronic structure and the geometry were 10^{-4} eV and 0.02 eV/Å, respectively. To improve the convergence of states near the Fermi level, we used the Gaussian smearing method with a finite temperature width of 0.2 eV.

2.8 Statistical Analysis

1. Pre-processing of data

Pre-processing data of Fig. 2A

1 layer GTO

Strain (%)	Parallel	$(R-R_o)/R_o$	Perpendicular	$(R-R_o)/R_o$
0	82	0	83	0
10	82	0	83	0
20	84	0.025	85	0.025
30	104	0.275	110	0.325
40	121	0.475	113	0.38
50	244	1.975	288	2.6
60	773	8.425	534	5.51
70	1107	12.5	950	10.45

Pre-processing data of Fig. 3E and 3F

1 layer GTO

Strain (%)	Parallel	(R-R0)/R0	Perpendicular	(R-R0)/R0
0	82	0	83	0
10	82	0	83	0
20	84	0.025	85	0.025
30	104	0.275	110	0.325
40	121	0.475	113	0.38
50	244	1.975	288	2.6
60	773	8.425	534	5.51
70	1107	12.5	950	10.45

2 layers GTO

Strain (%)	Parallel	(R-R0)/R0	Perpendicular	(R-R0)/R0
0	40	0	40	0
10	40	0	40	0
20	41	0.025	41	0.025
30	51	0.275	51	0.275
40	59	0.475	59	0.475
50	118	1.950	103	1.575
60	376	8.425	216	4.4
70	511	11.8	360	8

3 layers GTO

Strain (%)	Parallel	(R-R0)/R0	Perpendicular	(R-R0)/R0
0	15	0	15	0
10	15	0	15	0
20	15	0	15	0
30	19	0.267	19	0.267
40	22	0.467	22	0.467
50	37	1.466	31	1.06
60	113	7.5	77	4.13
70	169	10.3	130	7.7
80	240	15	195	12

4 layers GTO

Strain (%)	Parallel	(R-R0)/R0	Perpendicular	(R-R0)/R0
0	8	0	8	0
10	8	0	8	0
20	8	0	8	0
30	9.6	0.2	9.6	0.2
40	11.3	0.415	11.3	0.415
50	19	1.35	16	1
60	59	6	41	4.126

Pre-processing data of Fig. 3G (at 10^4 cycles)

1 layer GTO

Strain (%)	Parallel	$(R-R_0)/R_0$
0	82	0
30	271	2.3
40	615	6.5
50	1002	10
60	1394	16

2 layers GTO

Strain (%)	Parallel	$(R-R_0)/R_0$
0	40	0
30	132	2.3
40	260	5.5
50	400	9
60	640	15

3 layers GTO

Strain (%)	Parallel	$(R-R_0)/R_0$
0	15	0
30	42	1.8
40	97.5	5
50	135	8
60	210	13

4 layers GTO

Strain (%)	Parallel	$(R-R_0)/R_0$
0	8	0
30	20	1.5
40	44	4.5
50	56	6
60	88	10

2. Data presentation and sample size

1) **The estimated intensity ratio of I_{2D}/I_G and I_D/I_G:** The error ranges were determined using Raman beam size of 3 μm, measurement frequency of 20 times at 500 μm intervals, and beam intensity of 100 mW in sample size of 2×2 cm^2.

2) **The error ranges of sheet resistance:** The error ranges were determined by

measurements of 20 times in sample size of 2 × 2 cm^2.

3. ***Statistical methods for determination of graphene domain size and software:*** Domain size and distribution were determined by measurements above 80 domains in 2 × 2 cm^2 sample size. Determination of average domain size was performed by a standard deviation method using Excel File of computer.

References

[46] S. Doniach, M. Šunjić, *J. Phys. C: Solid St. Phys.* **1970**, 3, 285-291.

[47] B. Jang, C. H. Kim, S. T. Choi, K. S. Kim, K. S. Kim, H. J. Lee, S. Cho, J. H. Ahn and J. H. Kim, *2D Mater*. **2017**, 4, 024002.

[48] G. Kresse and J. Furthmuller, *Phys. Rev. B*, **1996**, 54, 11169–11186.

[49] J. P. Perdew, K. Burke and M. Ernzerhof, *Phys. Rev. Lett.* **1996**, 77, 3865–3868.

[50] P. E. Blöchl, *Phys. Rev. B*, **1994**, 50, 17953–17979.

Videos

Video S1 Demonstration for the outstanding performance of GTO/PDMS as a stretchable electrode by a LED switching test.

Video S2 Demonstration of stretchable LED control units by GTO-FETs. LED tests were performed under relaxed and 140% parallel stretchable strain to current direction.
